# The Organelle-Specific Regulations and Epigenetic Regulators in Ferroptosis

**DOI:** 10.3389/fphar.2022.905501

**Published:** 2022-06-17

**Authors:** Yixuan Zhang, Mingrui Li, Yiming Guo, Shuang Liu, Yongguang Tao

**Affiliations:** ^1^ Hunan Key Laboratory of Cancer Metabolism, Hunan Cancer Hospital and The Affiliated Cancer Hospital of Xiangya School of Medicine, Central South University, Changsha, China; ^2^ Department of Oncology, Institute of Medical Sciences, National Clinical Research Center for Geriatric Disorders, Xiangya Hospital, Central South University, Changsha, China; ^3^ NHC Key Laboratory of Carcinogenesis, Cancer Research Institute and School of Basic Medicine, Central South University, Changsha, China; ^4^ Key Laboratory of Carcinogenesis and Cancer Invasion, Ministry of Education, Department of Pathology, Xiangya Hospital, School of Basic Medicine, Central South University, Changsha, China; ^5^ Hunan Key Laboratory of Early Diagnosis and Precision Therapy in Lung Cancer, Department of Thoracic Surgery, Second Xiangya Hospital, Central South University, Changsha, China

**Keywords:** ferroptosis, epigenetics, cancer, organelles, post-translational modification, metabolism

## Abstract

Ferroptosis is fairly different from other types of cell-death in biochemical processes, morphological changes and genetics as a special programmed cell-death. Here we summarize the current literatures on ferroptosis, including the cascade reaction of key material metabolism in the process, dysfunction of organelles, the relationship between different organelles and the way positive and negative key regulatory factors to affect ferroptosis in the epigenetic level. Based on material metabolism or epigenetic regulation, it is obvious that the regulatory network of ferroptosis is interrelated and complex.

## 1 Introduction

Cell death is the universal outcome of cells. In the 1970s, cell death was segmented into apoptosis (type I), autophagy (type II) and necrosis (type III) on the basis of the early classification of cell morphology (Kroemer et al., 2005). In 2012, The Nomenclature Committee on Cell Death (NCCD) recommended molecular events in the death process replace cell morphology as a defining distinction (Kroemer et al., 2005). Compared with type III cell necrosis, the death process of type I and II is more orderly and complexly regulated. After the concept of programmed death was put forward, apoptosis and programmed death are often regarded as the same way. However, nowadays, people have gradually discovered that programmed death also includes some different death modes that do not rely on caspase like apoptosis, including ferroptosis. Ferroptosis was formally come up in 2012, used to describe RSLs-induced programmed cell death different from apoptosis (Dixon et al., 2012), depending on the iron and characterized by the reactive oxygen species (ROS) accumulating (Li et al., 2020). Different from necroptosis mediated by the combination of death receptor and death receptor ligand (Liu X. et al., 2021), the key central links of ferroptosis are iron-overload and ROS accumulation. First, the iron accumulating. Iron metabolism disorder causes Fenton reaction in cells, and then produces a mass of reactive oxygen species. The second stage is the imbalance of antioxidant system induced by the increasing of ROS (Leng et al., 2021). For such a new type of cell death that has many differences from the traditional way of death in biochemical metabolism and the morphology, how ferroptosis is triggered, how ferroptosis is inhibited, and the relation between ferroptosis and others are being explored (Cao and Dixon, 2016). As regard to ferroptosis, we will discuss and summarize its metabolic regulatory pathways, and how to affect the functional structure of organelles, its important key regulatory factors and epigenetic regulation pathways.

## 2 Regulation of Metabolic Pathways in Ferroptosis

### 2.1 Iron Metabolism

*In vivo*, iron is a vital metal element which is involved in biosynthesis and enzymatic reaction. Iron in human body is often divided into two types, functional iron (such as hemoglobin iron, transferrin iron) and stored iron (such as ferritin, hemosiderin). Firstly, Fe^2+^ is usually the initial way that enters into vivo, and is absorbed in the small intestine. After being oxidized by ceruloplasmin, it becomes Fe^3+^ iron that is easy to transport and binds to intracellular transferrin (TF) (Fuqua et al., 2012) to form TF-[Fe^3+^]_2_-TFR1, which is swallowed into the cells through the transferrin receptor 1 (TFR1) on the cell membrane. In the cells, TF-[Fe^3+^]_2_-TFR1 was reduced by iron reductase six-transmembrane epithelial antigen of prostate 3 (STEAP3) in lysosomes, and gathered in the intracellular labile iron pool (LIP) through the divalent metal transporter 1 (DMT1) (Torti and Torti, 2013). Then, Fe^2+^ can participate in different physiological processes, like the formation of functional enzymes, binding to ferritin temporarily stored, or through ferroportin discharged cells back to the blood medium (Chen J. et al., 2022).

**FIGURE 1:**
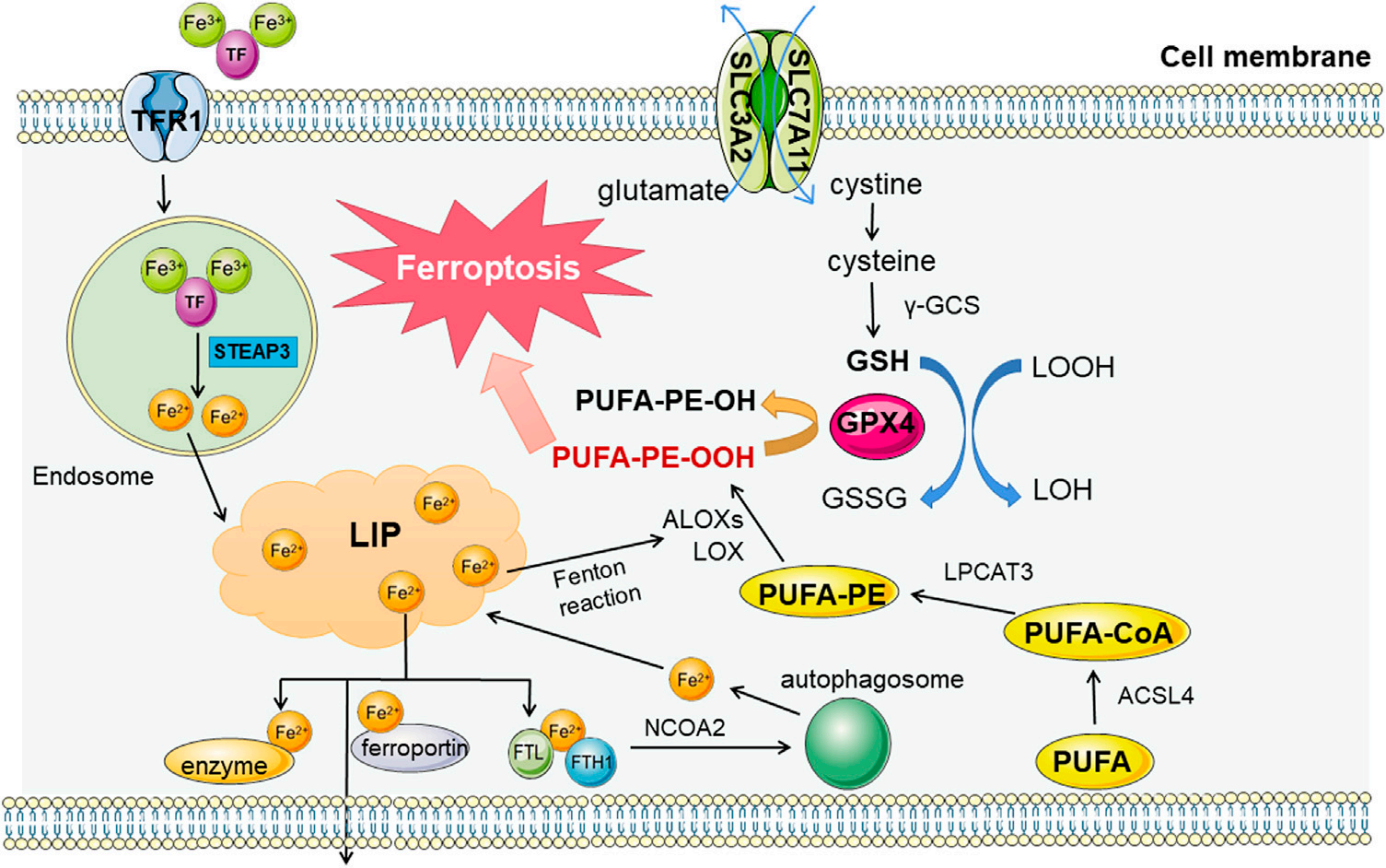
Regulation pathways in ferroptosis. There are three main metabolic pathways related to ferroptosis, iron metabolism, lipid metabolism and amino acid metabolism. The first two metabolites mainly promote the occurrence of ferroptosis, while the metabolites of amino acids (taking cysteine as an example) mainly play a role in resisting material peroxidation and ferroptosis. Fe^3+^ enters the cell through transferrin receptor 1 (TFR1) and is reduced to Fe^2+^ in lysosome. After entering the free iron pool, it can promote the formation and destruction of lipid peroxides, and hinder the removal of iron (including synthase, expelling and binding to ferritin storage) can lead to the overload of free iron. In addition, lipid peroxidation products are affected by Acyl-CoA synthetase long-chain family member 4 (ACSL4), lysophosphatidylcholine acyltransferase 3 (LPCAT3), Arachidonic acid lipoxygenases (ALOXS) and other enzymes, and are also catalyzed by Fe2+ Fenton reaction. Lipid peroxides produced have a central position in the process of ferroptosis. As a representative of antioxidant capacity, cysteine metabolic pathway can reduce lipid peroxides by Reduced glutathione (GSH). The coordination of the three maintain the stability of the internal environment, and the collapse of the balance will lead to the cascade reaction of ferroptosis.

Iron metabolic abnormalities *in vivo* is mainly due to abnormal iron input and discharge obstacles, triggering free iron accumulation in LIP. Then it leads Fenton reaction to generate lots of ROS, inducing ferroptosis (Liu J. et al., 2021). Ferroptosis inducers erastin or RSL3 can inhibit the antioxidant system and induce ferroptosis by promoting iron accumulation, and a variety of components mediating iron transport and utilization have been confirmed to affect the process of ferroptosis through regulation (Figure 1).

At the same time, as a storage unit, ferritin can also be degraded by autophagy mediated by nuclear receptor coactivator 4 (NCOA4), leading to the substantial increase in free iron (Hou et al., 2016) and subsequent cascade reactions. And the sensitivity to ferroptosis reflected in the earliest RAS-deficient cells of concern is also related to the subunits of ferroptosis (Yang and Stockwell, 2008).

### 2.2 Lipid Metabolism

Another important link of ferroptosis is lipid peroxidation. The Fenton reaction induced by iron overload can cause a large amount of ROS accumulation, and the free radicals in ROS can react with intracellular lipids to obtain a hydrogen atom from the allyl carbon in the lipid structure, forming lipid peroxide (ROOH) and generating a new free radical concurrently, and continuously circulating (Gaschler and Stockwell, 2017). In this process, the continuously-generated lipid peroxides are toxic, reflecting in two aspects-one is the destruction of biofilm. As an important component of the membrane, after lipid peroxidation, the membrane’s physical properties as a biofilm and cell recognition function will be affected (Catalá and Díaz, 2016), resulting in a series of physiological disorders of cells. On the other hand, lipid peroxide itself and its degradation products are also toxic (Kappus, 1987). Lipidomics has proved that polyunsaturated fatty acids (PUFA) can be preferentially oxidized by lipid peroxides, and this feature gives PUFA an important position in lipid metabolism related to ferroptosis. And similarly, the supplementation of PUFA can delay the ferroptosis process to a certain extent (Yang et al., 2016). This also shows to some extent that the content of PUFA can affect the degree of lipid peroxidation and determine the sensitivity of cells to ferroptosis (Magtanong et al., 2019).

Arachidonic acid (AA) and adrenaline (AdA), ω-6 fatty acids, are also the PUFAs most prone to lipid peroxidation (Tarangelo and Dixon, 2019). AA/AdA is converted into PUFA-CoA after acyl-CoA synthetase long-chain family member 4 (ACSL4) is added with an acyl group, and then it is converted into polyunsaturated fatty acids-phosphatidylethanolamine (PUFA-PEs) through lysophosphatidylcholine acyltransferase 3 (LPCAT3) catalysis (Zhang H. et al., 2021). PUFA-PES, an important substrate in the lipid peroxidation pathway, is oxidized to PUFA-PE-OOH under the action of lipoxygenase (ALOX) as a lipid peroxide to drive the ferroptosis process (Capelletti et al., 2020). This oxidation of ALOX can be enhanced by the Fenton reaction product of free iron and reversed by the important antioxidant component GPX4 in the amino acid metabolic pathway mentioned later (Xu et al., 2021), indicating the balance regulation function between different metabolic pathways.

ALOX is not only involved in lipid peroxidation, but also can be used as the target of classical ferroptosis regulators such as p53 for systematic regulation (Chu et al., 2019). Significant down-regulation of ALOX inhibits erastin-induced and RSL3-induced ferroptosis, and accordingly up-regulation of ALOX accelerates ferroptosis (Shintoku et al., 2017). While ACSL4 is involved in driving ferroptosis, it can also be used as a biomarker for ferroptosis (Yuan et al., 2016). Similarly, cytochrome P450 oxidoreductases with similar functions can also scavenge excessive lipid peroxides (Zou et al., 2020). These studies suggest that lipid synthase can not only adjust lipid oxidation but also take part in other links involved in ferroptosis to influent the cell-death progress.

### 2.3 Amino Acid Metabolism

GSH, composed of glutamic acid, has a significant application in the oxidation resistance ways against ferroptosis (Kuang et al., 2020). Cysteine, as a precursor of GSH, is also participating in the resistance to cell oxidation. On the one hand, it has its own antioxidant effect, on the other hand, the conversion of cystine to cysteine will affect the synthesis of glutathione (GSH) as a rate-limiting step (Lu, 2009). Transporter system Xc-can output glutamate in exchange for cystine accumulation by obtaining sufficient cystine to ensure intracellular cysteine supply for GSH synthesis (Bannai, 1986). It consists of solute carrier family three member 2 (SLC3A2) and solute carrier family seven member 11 (SLC7A11), of which SLC7A11 plays an active key role in ferroptosis (Sato et al., 1999).

Another vital antioxidant component of ferroptosis called glutathione peroxidase (GPX), a family of enzymes, worked as a cofactor of GSH to reduce excessive intracellular peroxides. However, only glutathione peroxidase 4 (GPX4) has a reduction effect on lipid peroxides (Forcina and Dixon, 2019), so the liveness of GPX4 is the basic of anti-lipid peroxide ability in ferroptosis (Ursini and Maiorino, 2020).

The collapse of this antioxidant line of defense is often a significant part of the ferroptosis process. The system Xc-responsible for input is often up-regulated in cancer cells due to the excessive demand for nutrients, but this effect can be blocked by the classical ferroptosis inducer erastin. The inhibition of erastin on SLC7A11 causes the depletion of intracellular cysteine, thereby affecting the production of GSH and the function of GPX4, which will lead to the disorder of antioxidant program in cells (Wang L. et al., 2020). In addition to the inhibition of system Xc-from the source, some small molecular inhibition and direct binding inhibition with GPX4 can also trigger ferroptosis. The target of ferroptosis inducer (1S, 3R) -RSL3 is GPX4, which can induce ferroptosis by inhibiting its phospholipid peroxidase activity (Yang et al., 2014). These studies indicate that the lack or depletion of nutrients, especially amino acids, has adverse effects on cells. Reducing the number of cells through the ferroptosis pathway may be the spontaneous choice of cancer cells under such adverse conditions, and can also provide new ideas for cancer treatment.

## 3 Involvement of Organelles in Ferroptosis

### 3.1 Ferroptosis in Mitochondria

**FIGURE 2:**
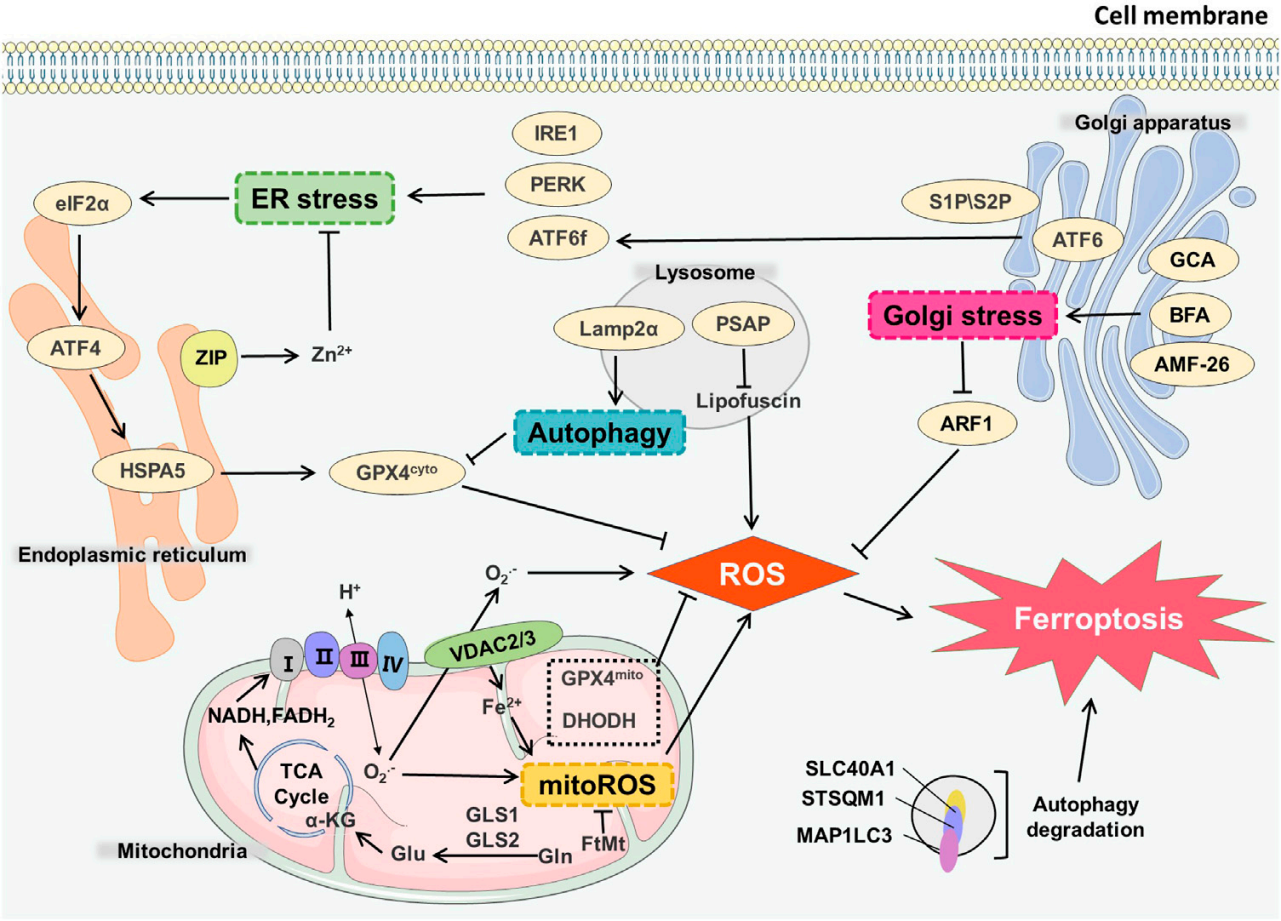
Organelles and ferroptosis. GLS1/GLS2 catalyzes Gln in the mitochondrial matrix to generate Glu, which is involved in TCA cycle to produce NADH and FADH2. After ETC, peroxides and mitoROS were generated. VDAC2/3 on the mitochondrial membrane mediated the maintenance of the steady state of Fe^2+^ in mitochondria, which also affected the generation process of mitoROS, and ultimately led to ferroptosis. GCA, BFA, AMF-26 induce Golgi stress and affect ferroptosis. Endoplasmic reticulum stress receptors such as PERK, ATF6, IRE1 can also cause endoplasmic reticulum stress-mediated ferroptosis. Autophagy degradation in lysosomes directly infulences the occurrence of ferroptosis. Lamp2α can inhibit GPX4 by affecting autophagy process, thereby increasing the level of ROS. PSAP can also affect lipofuscin and promote ROS generation.

Mitochondria is an important organelle for energy metabolism and ATP production in cells. There is a substantial connection between the generation of oxygen free radicals in mitochondria and programmed cell death (Liu and Shi, 2020). The function of the molecules and proteins in mitochondria in the process of ferroptosis has long been indeterminate. In ferroptosis cells, mitochondria often show significant morphological changes, including mitochondrial enlargement and crista disappearance (Doll et al., 2017), and the mitochondrial membrane potential abnormality may happen (Maru et al., 2022), suggesting that changes in normal mitochondrial function are closely associated with ferroptosis. Obviously, some mitochondrial-targeting chemicals have good inhibitory effect on ferroptosis (Krainz et al., 2016) (Figure 2).

The metabolism in mitochondria is the predominant source of reactive oxygen species (ROS) in cells. Complex II and complex III exist in mitochondrial inner membrane. The electrons produced in oxidative phosphorylation can leak and produce superoxide (O_2_·-) through complex II and complex III. Under the catalysis of superoxide dismutase (SOD), O_2_—disproportionately reacts to produce H_2_O_2_ and further generates OH through the Fenton reaction. The production of ROS affects the normal function of mitochondria and the redox state of other organelles (Zhang B. et al., 2022). System Xc-is a transporter presenting on the cell membrane, cystine import by system Xc-is simultaneously accompanied by glutamine export. The decomposition of glutamine can produce α-ketoglutarate, which participates in the tricarboxylic acid cycle (TCA) cycle occurring in the mitochondrial matrix and mimic the death-inducing activity of L-Gln, thereby regulating the ferroptosis (Gao et al., 2015). Suppression of TCA cycle or electron transfer chain (ETC) can effectively reduce the production of lipid peroxides (Zhao et al., 2022), thereby reducing the occurrence of ferroptosis, indicating that ferroptosis is related to abnormal ROS production. At the same time, glutamine decomposition can generate intermediates of the TCA cycle which induces mitochondrial membrane hyperpolarization, and it may reflect the increase of ETC activity and ROS generation (Tang E. et al., 2021). Mitochondrial membrane hyperpolarization may reflect increased ETC activity and ROS generation (Bonanno et al., 2022).

GPX4 is one of the most important enzymes involved in ferroptosis, and it weakens the toxicity of lipid peroxides to maintain the homeostasis of phospholipid bilayers and resist oxidative damage. However, during the ferroptosis induced by GPX4 inhibition, mitochondria is non-essential (Gao et al., 2019). Some enzymes, such as monoamine oxidase and α-ketoglutarate dehydrogenase, in the catalytic process can also produce ROS (Adam-Vizi and Tretter, 2013). Coenzyme Q10 (CoQ10), a regulator of mitochondrial permeability transition pore (mPTP) as a key part of ETC, inhibits the occurrence of ferroptosis (Ernster and Forsmark-Andrée, 1993). Myristylation recruits ferroptosis suppressor protein 1 (FSP1) to mediate the reduction of NADH-dependent CoQ10, and this process inhibits the transmission of lipid peroxides to prevent ferroptosis. Exogenous supplement of CoQ10 can also protect cells from oxidative stress (Bersuker et al., 2019; Rizzardi et al., 2021).

Mitochondria are closely involved in the formation of intracellular iron homeostasis. The free state and redox state of intracellular iron affect the occurrence of ferroptosis. Voltage-dependent anion channels (VDAC) participate in the regulation of the transport of iron in mitochondria, and erastin destroys the expression of VDAC2 and VDAC3 (Yagoda et al., 2007). Down-regulation of mitochondrial VDACs can also effectively inhibit ferroptosis (Yang and Stockwell, 2008). Mitochondrial ferritin (FtMt) controls the size of mitochondrial LIP and attenuates the level of ROS. Also FtMt can significantly inhibit ferroptosis induced by erastin, and maintain the level of Bcl-2 and mitochondrial membrane potential to diminish caspase three expression (Wang YQ. et al., 2016; Gao et al., 2017).

Dihydroorotate dehydrogenase (DHODH) is an enzyme expressed in the mitochondrial inner membrane, which catalyzes the *de novo* synthesis of pyrimidine nucleotides, the deletion of DHODH can induce cell death in cells with high GPX4 expression without causing lipid peroxidation, and this process can be rescued by supplementing uridine (Vasan et al., 2020). In cell lines with low GPX4 expression such as NCI-H226, even when supplemented with uridine, DHODH can still induce lipid peroxidation and ferroptosis. The cell lines could not be cultured for a long time, indicating that DHODH acts in parallel to GPX4 in ferroptosis whereas the inhibition of DHODH has no impact on the expression of GPX4. It also suppresses lipid peroxidation by reducing CoQ to CoQH2, suggesting that DHODH and mitochondrial GPX4 constitute the two main defense mechanisms of lipid peroxide detoxification in mitochondria (Mao et al., 2021).

However, there is also some evidence against the main role of mitochondria in ferroptosis. Cell lines lacking mitochondrial DNA (ρ0 cell lines) have no significant difference in ferroptosis sensitivity compared with their parental cell lines. By evaluating the distribution of ferrostatin-1 and ferrostatins in organelles, it is found that mitochondria are not necessary to inhibit ferroptosis (Gaschler et al., 2018). Therefore, to further investigate the role of mitochondrial metabolism in ferroptosis model is necessary, and to clarify whether mitochondrial and mitochondrial-related signaling pathways can be used as effective targets for regulating ferroptosis.

### 3.2 Ferroptosis in Endoplasmic Reticulum

Endoplasmic reticulum (ER) is a complex membrane system performing the functions of protein, lipid and carbohydrate synthesis. The ER is spatially and functionally connected to other organelles such as the mitochondria, lysosomal system, etc (Daniele and Schiaffino, 2014). The stability of endoplasmic reticulum structure and function directly affects the normal function of other organelles. ER stress occurs when the cells deal with the misfolded or unfolded proteins in endoplasmic reticulum as well as calcium ion balance disorders (Lei et al., 2022). For example, unfolded protein response (UPR) can be initiated through three signaling pathways, separately mediated by three transmembrane ER receptors, AMP-dependent transcription factor (ATF6), inosital-requiring enzyme-1 (IRE1) and protein kinase R-like ER kinase (PERK) (Zanetti et al., 2022). Amino-terminal cytoplasmic fragment (ATF6f) produced by the cleavage of ATF6 is involved in mediating ER protein folding. IRE1 oligomerization forms IRE1 RNase, inducing regulated IRE1-dependent decay (RIDD). ATF6 phosphorylates eukaryotic translation initiation factor 2 (eIF2α) and up-regulates ATF4, ATF4 controls the expression of related genes such as C/EBP-homologous protein (CHOP) (McGrath et al., 2021).

ER stress is closely related to ferroptosis in many cases, such as acute kidney injury (Li et al., 2022), type II diabetes (Sha et al., 2021), and tumors (Sun et al., 2015; Zhu et al., 2017). Several lines of evidence highlight that erastin can induce ER stress through the activation of eIF2α/ATF4 signaling pathway (Dixon et al., 2014), while sorafenib can activate IRE1 and p-ERK, but the activation of p-ERK has a protective effect on cells (Rahmani et al., 2007). Dihydroartemisinin (DHA) can activate ATF4 by promoting the level of p-ERK in glioma cells, thereby increasing the level of heat shock protein family A (Hsp70) member five HSPA5. HSPA5 has the effect of up-regulating the GPX4, which results in protecting glioma cells from ferroptosis, indicating the activation of ATF4 strengthens the drug resistance of glioma (Chen Y. et al., 2019). p-ERK-mediated ATF4 activation can also protect pancreatic cancer cells from ferroptosis (Dixon et al., 2014), and evidence shows that cystine starvation can increase the phosphorylation of eIF2α, as well as the expression of ATF4, in human triple negative breast cancer (TNBC) cells, resulting in ferroptosis (Chen et al., 2017).

There are other pathways besides the ER stress that affect the occurrence of ferroptosis. For example, ZIP7 controls zinc transport from the ER to the cytoplasm (MacKenzie and Bergdahl, 2022), a reduction in ZIP7 expression will cause ferroptosis protection. This process may be related to the involvement of zinc in ER and nuclear communication (Chen PH. et al., 2021). Exogenous monounsaturated fatty acids (MUFA) can reduce the production of lipid peroxides on ER membrane, and this ACSL3-dependent process induces ferroptosis-resistance (Magtanong et al., 2019).

ER has a certain contact with other organelles. ER and mitochondria can be linked through mitochondrial-associated ER membrane (MAM) (Means and Katz, 2021). Hence, MAM is essential for the maintenance of intracellular calcium homeostasis and cell survival (Lee and Min, 2018). Therefore, the future study should give attention to the more precise delineation of the signal network between endoplasmic reticulum and other organelles.

### 3.3 Ferroptosis in Golgi Apparatus

This membranous organelle is involved in the secretory function and glycosylation of proteins. Golgi disruptors, including brefeldin A (BFA), golgicide A (GCA), and 2-methylcoprophilinamide (AMF-26, also called M-COPA), can act on Golgi to cause oxidative stress. The production of superoxide and peroxide induces ferroptosis. The lipid peroxides accumulate after Golgi stress happens, which also lessen the intracellular glutathione. Antioxidants, reactive oxygen species scavengers and GPX4 can promote ferroptosis-resistant cells stage induced in Golgi stress, indicating that reducing the production of lipid peroxides is beneficial to maintain the steady state of secretion pathway (Alborzinia et al., 2018). Golgi-associated small GTPase ADP ribosylation factor 1 (ARF1) can reduce the production of lipid peroxides. When Golgi stressors and low doses of ferroptosis inducers are applied at the same time, ferroptosis inducers can significantly reduce the effect of the Golgi stressors on inducing cell death (Alborzinia et al., 2018). This process may be related to the common antioxidant regulation and feedback mechanism such as the transsulfuration pathway initiated by cells, which enhance viability upon Golgi stressors (Alborzinia et al., 2018). Therefore, it is speculated that during ferroptosis, the normal function of Golgi apparatus is affected, resulting in the destruction of cell secretion and the steady state of lipid ROS generation.

### 3.4 Ferroptosis in Lysosome

Autophagy, a lysosomal degradation pathway that degrades ferritin in cells, induces ferroptosis (Hou et al., 2016; Kang and Tang, 2017). Blocking the function of cathepsin and H^+^-ATPase can limit ferroptosis (Gao et al., 2018). Ferroptosis often occurs when the function of GPX4 is inhibited or insufficient. Studies have shown that erastin increases the expression of lysosome-associated membrane protein 2α (Lamp2α), which promotes molecular chaperone-mediated autophagy and down-regulate GPX4 (Wu et al., 2019). Following the treatment of cells with lysosome activity inhibitors, the outbreaks of lysosome ROS are significantly reduced, and the lipid peroxides in the perinuclear space are also significantly attenuated, suggesting that lysosomes may participate in ferroptosis by regulating ROS generation (Torii et al., 2016; Kumar et al., 2021).

Ferroptosis activators (erastin and RSL3) decrease GPX4 expression in human tumor cell lines. RSL3 up-regulates the microtubule-associated protein one light chain three beta (MAP1LC3B-II) or down-regulates sequestosome 1 (SQSTM1) (Liu J. et al., 2019). Accordingly, it increases the level of intracellular iron ions and promotes the occurrence of ferroptosis (Li et al., 2021). Furthermore, autophagy degradation of intracellular lipid droplets is conducive to the accumulation of free fatty acids, promoting ferroptosis in RSL3-induced cells (Bai et al., 2019).

The targeted fluorescent probe used in erastin-treated HT1080 cells shows the intracellular iron homeostasis has been changed, and the increase of unstable Fe2+ in lysosomes leads to ferroptosis (Hirayama et al., 2019). The decrease in the expression of lysosomal protein prosaposin (PSAP) could trigger the formation of lipofuscin, which promotes the accumulation of iron and ROS, leading to ferroptosis (Tian et al., 2021). Studies have also shown that NTP-activated ringer‘s lactate (PAL) can cause NO-mediated lysosomal damage, triggering ferroptosis in malignant mesothelioma cells (Jiang et al., 2021b). Recent insights show that ferroptosis is dependent on autophagy, which is a lysosome-mediated process of recycling cellular components. Therefore, future studies can further explore the autophagy-based induction of ferroptosis in tumor cells.

## 4 Key Regulators of Ferroptosis

### 4.1 Transcription Factor NRF2

**FIGURE 3:**
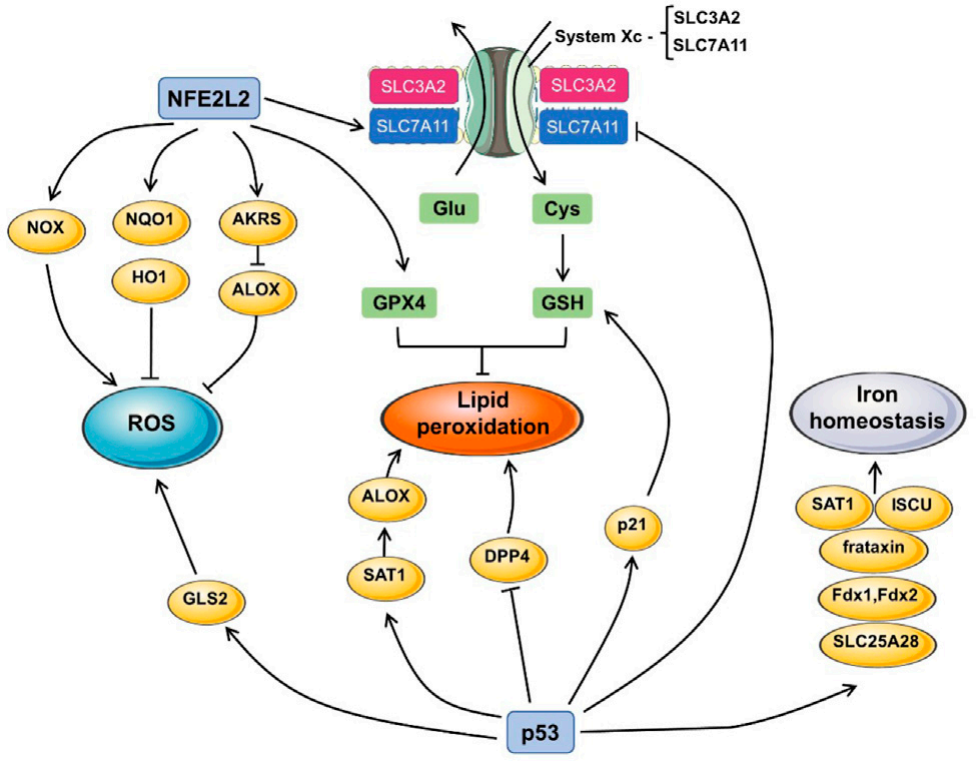
The pathway of p53 and Nrf2 in ferroptosis. The generation of ROS, lipid peroxidation and iron homeostasis are the indispensable course in triggering ferroptosis. Nrf2 and p53 participate in ferroptosis by influencing the expression of a series of downstream proteins. For example, Nrf2 promotes the expression of GPX4 and upregulates the expression or the function of solute carrier family seven member 11 (SLC7A11) to exert influence on lipid peroxidation. Nrf2 also activates NADPH oxidases (NOX), NAD(P)H quinone dehydrogenase 1 (NQO1), heme oxygenase 1 (HO1), aldosterone reductases (AKRS), which all contribute to the production of ROS, resulting in ferroptosis. GLS2 induced by p53 inhibit the depletion of ROS. P53 also promote the synthesis of Spermidine/spermine N1-acetyltransferase 1 (SAT1) and dipeptidyl peptidase 4 (DPP4), which enhance the lipid peroxidation. P53 also regulates frataxin, ferredoxin one and 2 (Fdx1/Fdx2), iron-sulfur cluster assembly enzyme (ISCU), SAT1, the activation of those molecules contributes to iron homeostasis.

Nrf2 is an essential transcription factor with antioxidant function, and its content remains at a low level through three different E3—ubiquitin ligase complexes, Kelch-like ECH-associated protein 1-Cullin 3-Ring box 1 (KEAP1-CUL3-RBX1), (SKP1, CUL1, and F-box protein)/β-Transducin repeats-containing protein (SCF/β-TrCP) and synovin/Hrd1 (Li et al., 2019; Wu et al., 2020). Kelch ECH-associated protein 1 (KEAP1) is one of the main negative regulatory factors of Nrf2 (Bellezza et al., 2018). Normally, KEAP1 regulates NRF2 activity by targeting ubiquitination and proteasome-dependent degradation, which interact and remain in the cytoplasm (Huang et al., 2010; Baird and Yamamoto, 2020). Under oxidative stress, the cysteine residues of KEAP1 were modified and then lost the ability to degrade Nrf2 (Taguchi et al., 2011). Nrf2 dissociates from Keap1 and accumulates in the nucleus to activate other protective genes in anti-oxidative stress response (Deshmukh et al., 2017) (Figure 3).

Given the role of NRF2 in multiple key pathways against oxidative stress, it is not difficult to imagine the effect of Nrf2 on iron cell death. For the first time in hepatocellular carcinoma (HCC), researches have demonstrated the effectiveness of Nrf2 in protecting cells from ferroptosis, this pathway can also be used by ferroptosis activators including sorafenib, which stabilizes NFE2L2 in human HCC cells by inhibiting the formation of NFE2L2-KRAP1 complexes. This process is further regulated by SQSTM1/p62. P62, a kind of ubiquitin-binding protein, competes with NRF2 in the Kelch motif of KEAP1 and increases the content of downstream Nrf2 by inactivation of Keap1. It also shows that the regulation of Nrf2 by KEAP1 will be competitively interfered by many KEAP1-binding proteins (Sun et al., 2016; Nam and Keum, 2019).

There are many downstream genes of Nrf2, which mainly inhibit the occurrence of ferroptosis through several groups of targeted genes with different targets, including iron metabolism, intermediate metabolism and GSH synthesis/metabolism, and no matter what kind of mode, they all involve antioxidant reaction elements (ARE) (Rojo de la Vega et al., 2018; Dodson et al., 2019). As a typical anti-oxidative stress molecule, the enhanced Nrf2 can upregulate multiple ROS inhibitor (Guo et al., 2021), thereby protecting cells from oxidative stress. In iron metabolism, existing studies have found that Nrf2-regulated genes involved in iron metabolism can affect several links including heme synthesis, hemoglobin catabolism, iron storage and iron output (Kerins and Ooi, 2018). Nrf2 can regulate iron/heme metabolism pathway, and Heme oxygenase 1 (HO1) is an enzyme catalyzing the conversion of heme to choline which is related to it (Balla et al., 2007; Kerins and Ooi, 2018). HO-1, a targeted gene of Nrf2 participates in the induction of ferroptosis and accelerates erastin-induced ferroptosis (Kwon et al., 2015; Chang et al., 2018). Nrf2-HO-1 pathway also has moderating effect on intracellular iron concentration (LIP) (Tomiotto-Pellissier et al., 2018), but it needs further study to see whether this pathway is the main one to regulate free iron concentration. However, the role of HO-1 has both positive and negative regulatory directions, which is often concerned about the protective effect of HO-1 on cells. The specific regulatory direction may depend on the pathological state and internal environment of the cell itself.

In iron storage, ferritin is a protein formed of light chain (FTL) and heavy chain (FTH1) subunits (Muhoberac and Vidal, 2019), which participates in iron storage, which can also be affected by Nrf2 (Sun et al., 2016; Muhoberac and Vidal, 2019). As for the iron transport related heme transporter solute carrier family 48 member 1(SLC48A1), Nrf2 also plays an up-regulated role in SLC48A1’s participating in ferroptosis (Campbell et al., 2013).

In the intermediate metabolism of intracellular substances, NADPH is indispensable for the reduction and oxidation of substrates. NADPH, NADPH oxidase and its homologous NOX family are also affected by Nrf2 (Bedard and Krause, 2007). Nrf2 can produce ROS through NADPH oxidase, leading to the accumulation of ROS, which also plays a similar role in the NOX family. On the other hand, aldosterone reductases (AKRs) gene family (AKR1C1/3) is also regulated by Nrf2 (Gagliardi et al., 2019).

In terms of GSH metabolism, Nrf2 can regulate GSH production by affecting glutamate-cysteine ligase (GCL) and GCL-catalyzed/modified subunit (GCLC/GCLM). Also regulated by Nrf2 are the subunit SLC7A11 of cystine/glutamate transporter xCT, and GPX4 downstream of GSH, which exert a profound influence on the production and function of GSH (Fan et al., 2017; Shin et al., 2018; Jin et al., 2019).

### 4.2 TP53

Mutational activation of the TP53 is one of the most common genetic alteration in human cancer (Hoang and Landi, 2022). As an anti-oncogene, TP53 is involved in all aspects of normal physiological activities of cells, including the regulation of glycolysis and oxidative phosphorylation, and the regulation of ROS generation (Kruiswijk et al., 2015). Its translation product p53 is a specific DNA-binding protein with the function of transcription factors, which participates in various cell stress and cell cycle, and also mediates cell senescence and death (Laptenko and Prives, 2006). Ferroptosis is characterized by iron-dependent lipoperoxide accumulation, and p53 can participate in the occurrence of ferroptosis by affecting iron metabolism and lipid metabolism.

Iron homeostasis interacts with the expression of p53. Excessive iron could reduce p53 expression level, whereas the iron polyporphyrin heme could restrain the combination between p53 and DNA, promoting the export of p53 from the nucleus and degradation in the cytoplasm (Shen et al., 2014). After human lymphocytes were treated with deferoxamine (DFX), p53 increased but its downstream target protein was not affected (Kim et al., 2007). In addition, p53 can up-regulate the expression of frataxin, which affects the iron homeostasis in mitochondria and prevents the occurrence of iron overload (Shimizu et al., 2014). P53 also affects the expression of ferredoxin reductase (FDXR) (Zhang Y. et al., 2022). FDXR transfers the reduction equivalent obtained from NADPH to ferredoxin 1 and 2 (Fdx1, Fdx2) (Brandt and Vickery, 1992). Fdx1 is involved in steroid metabolism and the synthesis of iron sulfur protein. The limitation of Fdx2 function leads to increased iron uptake in cells and iron accumulation in mitochondria (Sheftel et al., 2010). P53 can also induce the expression of iron-sulfur cluster assembly enzyme (ISCU). ISCU inhibits the binding of iron regulatory protein 1 (IRP1) to ferritin heavy peptide 1 (FTH1) and promotes the translation of FTH1, which will increase intracellular iron storage (Funauchi et al., 2015).

P53 affects systemic lipid metabolism by promoting intestinal lipid absorption (Goldstein et al., 2012), and it also plays a vital role in intracellular lipid metabolism. P53 can bind with glucose-6-phosphate dehydrogenase to inhibit NADPH formation. Since NADPH is an important auxiliary factor in fatty acid synthesis, its synthesis limitation directly affects lipid synthesis (Jiang et al., 2011). P53 also affects sterol regulatory element binding protein-1(SREBP-1), regulating the lipid synthesis through the IGF-1-AKT-mTOR pathway (Yahagi et al., 2003; Feng and Levine, 2010).

In addition, p53 can inhibit the expression of solute carrier family seven member 11 (SLC7A11) (Ritzenthaler et al., 2022), SLC7A11 is an important part of the cystine/glutamate antiporter, so the decrease of SLC7A11 expression inhibits the cystine ingestion and sensitizes cells to ferroptosis (Jiang et al., 2015). Since SLC7A11 can specifically bind and inhibit ALOX12 (arachidonate 12-lipoxygenase, an enzyme in another pathway other than GPX4-mediated effect on intracellular lipoperoxide level, p53 indirectly affects the activity of ALOX12 and influences the lipoperoxide level in the cytoplasm (Kappus, 1987). Phosphate-activated glutaminase (GLS2) can promote glutamine decomposition and reduce intracellular ROS level, and P53 affects intracellular antioxidant capacity by regulating GLS2 expression (Suzuki et al., 2010; Shi et al., 2022). Another study showed that the decrease of p53 expression in colorectal cancer accumulates Dipeptidyl peptidase 4 (DPP4) in nuclear, and facilitates membrane lipid peroxidation and leads to ferroptosis (Xie et al., 2017). Polyamines are closely associated with the occurrence of cancer (Gerner and Meyskens, 2004). Spermidine/spermine N1-acetyltransferase 1 (SAT1) is a limiting enzyme that controls the metabolism of polyamines. P53 activation can significantly upregulate the expression level of SAT1. Cells with high expression of SAT1 die after ROS treatment. This process can be suppressed by ferroptosis inhibitor Ferrostatin-1. Meanwhile, the expression level of ALOX15 is promoted. After the SAT1 expression was knocked down in p53-induced ferroptosis, the expression of ALOX15 was attenuated. It is speculated that ALOX15 may be a downstream molecule of SAT1 (Ou et al., 2016). p53 forms a complex with soluble carrier family 25 member 28 (SLC25A28) and activates SLC25A28, affecting the function of ETC and iron homeostasis, leading to ferroptosis in hepatic stellate cells (HSCs) (Zhang et al., 2020). P21, a downstream molecule of p53, can also interact with CDK and enhance cell resistance to ferroptosis in some tumor cell lines (Venkatesh et al., 2020).

Since p53 has effects on the adjustment of different target genes expression under different environmental backgrounds, the role of p53 in the occurrence of ferroptosis is very complex. This most widely studied gene, can be a powerful target for future treatment in ferroptosis (Liu and Gu, 2022). The bidirectional control of ferroptosis is context-dependent, so the role and regulation of p53 in the occurrence of ferroptosis still need further study.

## 5 Epigenetic Remodelers in Ferroptosis

### 5.1 Non-Coding RNAs

Non-coding RNAs are divided into several categories, long non-coding (lnc) RNA NEAT1 regulates the sensitivity of non-small cell lung cancer (NSCLC) to iron-dependent lipid peroxide accumulation by affecting the acyl-CoA synthetase long-chain family member 4 (ACSL4), thereby affecting the resistance of cancer cells to ferroptosis (Wu and Liu, 2021). MiRNA-17-92 can also reduce the erastin-induced ferroptosis in endothelial cells with the help of the A20-ACSL4 axis (Xiao et al., 2019).

In the meantime, the mutual regulatory pathways of different types of non-coding RNAs often have value in ferroptosis. In rat pulmonary fibrosis tissues, silencing of lncRNA ZFAS1 affects miR-150-5p, resulting in down-regulation of SLC38A1, thereby attenuating lipid peroxidation and ferroptosis induced by bleomycin (BLM) and transforming growth factor-β1 (TGF-β1). lncRNA PVT1 in cerebral ischemia/reperfusion (I/R) can reduce the binding of miR-214 to PVT1 and TP53 3′UTR by restraining the expression of miR-214, thereby enhancing ferroptosis. This pathway has a positive feedback regulation mechanism, and different coding RNAs also interact in this process (Yang et al., 2020b; Lu et al., 2020). Chloramphenone can suppress lncPVT1, and weaken the effect of miR-214-3p on GPX4, thereby accelerating ferroptosis (He et al., 2021). lncRNA LINC00336, as a competitive endogenous RNA (ceRNA), combines with the ELAV-like RNA-binding protein 1 (ELAVL1). ELAVL1 can inhibit ferroptosis in lung cancer cells, and ELAVL1 itself also promotes this process by stabilizing LINC00336 transcription. LINC00336 can also regulate cystathionine-β-synthase (CBS) by affecting microRNA 6852 (MIR6852), and CBS can trigger the ferroptosis process of cancer cells (Wang et al., 2019). The ectopic expression of metallothionein 1D pseudogene (MT1DP) can increase the sensitivity of lung cancer cells to erastin-induced ferroptosis by down-regulating NRF2, while up-regulating MDA and ROS levels to promote ferroptosis (Gai et al., 2020).

While up-regulating caspase-3 to promote cell apoptosis, LINC00618 can also promote ferroptosis by increasing the free iron content and ROS accumulation in cells. This phenomenon that LINC00618 is regulated by lymph-specific helicase (LSH) confirms that the regulation of non-coding RNA in the field of ferroptosis can also be affected by other epigenetic pathways. In view of the key role of LSH in ferroptosis-related chromatin remodeling and DNA methylation modification pathway, it cannot be ignored (Wang Z. et al., 2021; Chen X. et al., 2022).

On the other hand, LSH can down-regulate the expression of lncP53RRA, and P53RRA can bind to Ras GTPase activator protein binding protein 1 (G3BP1). The decrease of P53RRA leads to the replacement of P53 from G3BP1, and P53 being retained in the nucleus more It will also results in subsequent cell cycle arrest and ferroptosis (Mao et al., 2018). The regulation of LSH on LINC00618 and P53RRA on ferroptosis pathway also reflects the cross of different epigenetic modification pathways to some extent.

A large number of lncRNAs not only play a role in ferroptosis, a considerable number of indicators can be used as prognostic factors for ferroptosis through the investigation of cancer genome map (TCGA) database. For example, 25 lncRNAs are found to be the independent prognostic factors in head and neck cancer (HNSCC) (Tang Y. et al., 2021).

### 5.2 Chromatin Remodeling Factors

Chromatin remodeling usually depends on the covalent modification of nucleosomes. Using ATP as the energy source, this process works in adjusting cell cycle progressing, cell movement and nuclear hormone signal transduction (Dawson and Kouzarides, 2012). Through genome sequence analysis, chromatin remodeling complex dysfunction is widespread in major cancers, and complex mutations and other genetic changes often occur (Zhang et al., 2016). LSH, as a chromatin remodeling protein, belongs to the chromatin remodeling ATPase SNF2 family, and generally plays a stabilizing role by maintaining an appropriate DNA methylation level (He et al., 2016; Jiang et al., 2017; Xiao et al., 2017; Chen L. et al., 2019; Liu et al., 2020; Chen X. et al., 2022). Studies have shown that LSH can also take part in the metabolic paths associated with ferroptosis to achieve regulatory purposes, except for the above-mentioned pathways that control non-coding RNAs to participate in ferroptosis. For example, LSH inhibits ferroptosis by up-regulating the enrichment of WD40 protein WDR76 (nuclear WD40 protein) on lipid metabolism-related genes, thereby reducing iron accumulation and lipid ROS load (Jiang et al., 2017). These studies show that chromatin remodeling mainly affects the expression level of ferroptosis-related genes through gene expression regulation mode, thereby regulating ferroptosis.

### 5.3 Post-Translational Modifications and Ferroptosis

#### 5.3.1 Protein Ubiquitination and Ferroptosis

Similarly, the resistance and induction of cells to ferroptosis are also related to the ubiquitination process. Neuronal precursor cell-expressed developmentally downregulated 4 (Nedd4), as a ubiquitin ligase of the conserved HECT domain E3, can promote voltage-dependent anion channel (VDAC) ubiquitination and degradation by unifying with VDAC, which can lead to ferroptosis resistance in the treatment with erastin, an ferroptosis inducer. The treatment of erastin can trigger the transcriptional activation of Nedd4 in melanoma cells, leading to the degrading of VDAC2/3, the target of erastin (Yang et al., 2020a). However, deubiquitinases have opposite effects on ferroptosis. For example, deubiquitinase ubiquitin hydrolase OTUB1 can maintain SLC7A11’s stability through direct interaction with it, and this process does not depend on the participation of P53 (Liu T. et al., 2019).

On the other hand, deubiquitinase can have different effects on ferroptosis. For example, deubiquitinase ubiquitin hydrolase OTUB1 can directly interact with and maintain the stability of SLC7A11, and this process does not depend on the participation of P53 (Liu T. et al., 2019).

Ubiquitination modification is common in various diseases, and the ubiquitination of key factors of ferroptosis is participate in the procedure of ferroptosis-related diseases like diabetic retinopathy (DR) (Zhang J. et al., 2021), impaired cognitive dysfunction (POCD) (Zhao et al., 2021) and ischemia/reperfusion (I/R) (Tang LJ. et al., 2021). Ubiquitination modification also widely exists in several major metabolic processes related to ferroptosis. In terms of iron metabolism, FPN1, the only iron-exporting protein on the cytoplasmic membrane, has been confirmed to be able to be ubiquitinated and thus affect its function. For example, ubiquitin ligase RNF217 mediates the degradation of iron-exporting protein FPN and regulates iron homeostasis (Jiang et al., 2021a). As for the lipid metabolism, tumor suppressor BRCA1-associated protein 1 (BAP1) reduces the binding rate of SLC7A11 promoter to H2A-ub, and inhibits SLC7A11 and cystine uptake by deubiquitinating enzyme (DUB) encoded by BAP1, contributing to lipid peroxidation and ferroptosis (Zhang et al., 2018). E3 ubiquitin ligase named Tripartite motif-containing protein 26 (TRIM26) can promote SLC7A11 ubiquitination and degradation in HCC to promote lipid peroxidation and ferroptosis downstream (Zhu et al., 2021). The latest development of mass spectrometry can qualitatively and quantitatively analyze the ubiquitination of cancer specimens, which has a positive significance for revealing the causes of abnormal protein expression of cancer genes (Faktor et al., 2019).

The ubiquitination process also has a significant effect on the ferroptosis’s key regulatory factors. For example, Nrf2, the main negative regulatory factor of ferroptosis, can be deubiquitinated by ubiquitin-specific-processing protease 11 (USP11) to increase the stability of its own proteins, so as to better participate in the process of ferroptosis (Meng et al., 2021). On the contrary, Quiescin sulfhydryl oxidase 1 (QSOX1) can indirectly inhibit the activity of Nrf2 by ubiquitinizing Epidermal growth factor receptor (EGFR) (Sun et al., 2021).

#### 5.3.2 Protein Methylation and Ferroptosis

As a dynamic regulation mechanism, protein methylation is widely present in histone and non-histone post-translational modifications (Hamamoto et al., 2015), which has considerable influence on cell cycle, apoptosis and cancer progression. More attention has been paid to histone methylation modification, such as histone H3 lysine nine demethylase B (KDM3B). SLC7A11, as one of the subunits of Xc-, can be used as a downstream factor of ferroptosis inhibitor. kDM3B can inhibit ferroptosis caused by Erastin, an ferroptosis inducer, by activating SLC7A11 (Wang Y. et al., 2020). Similarly, ferroptosis also take part in the pathway that SET domain bifurcated 1 (SETDB1) regulates epithelial-mesenchymal transition (EMT) in lung cancer. For cells without EMT, SETDB1 indirectly induces downstream E-cadherin downregulation by catalyzing the histone H3 lysine nine trimethylation (H3K9me3) of the main EMT transcription factor Snai1, thereby increasing ferroptosis (Liu et al., 2022). (+) -JQ1 can reduce the expression of histone lysine methyltransferase G9a by epigenetic way, thereby inhibiting Bromodomain-containing protein 4 (BRD4), a tumor-driven bromodomain protein that is often highly expressed in tumors, to regulate ferritin phagocytosis, iron accumulation and reactive oxygen species production (Sui et al., 2019).

For the modification of non-histone proteins, studies have shown that the methylation modification of non-histone proteins can regulate the process of cancer, and the specific effects vary according to different substrates (Wei et al., 2014). The role of non-histone methylation in ferroptosis and possible pathways needs to be further explored.

#### 5.3.3 Protein Acetylation

The lysine acetylation process takes effect in lots of biological phenomena, and one included is ferroptosis. Acetylation of P53 has a considerable effect on its influence on apoptosis and ferroptosis. Studies have shown that the acetylated mutant p53 3KR retains its iron-induced death function though it loses its ability to regulate cell cycle and other aspects (Jiang et al., 2015). The acetylation of proteins will affect functions, and the adjustment of the degree of acetylation will also have different effects, which partly depends on the site of acetylation. When the acetylation of K98 (p53 4KR98) on P53 is lost, there is only a slight functional effect. When K98 and K117/161/162 acetylation (p53 3KR) are eliminated simultaneously, the downstream function mediated by p53 is significantly inhibited, including downstream molecules such as GLS2 and SLC7A11 (Wang SJ. et al., 2016). Sodium hydrosulfide (NaHS) can significantly alleviate ferroptosis in prefrontal cortex (PFC) of diabetic mice. This is mainly because NaHS can decrease the iron accumulation and oxidative stress, and GPX4 and SLC7A11can also be enhanced by NaHS. In addition, the effect of NaHS on alleviating anxiety in diabetic mice is based on its inhibition of ferroptosis in central nervous cells, and also involves the participation of protein acetylation. NaHS can boost the sirtuin 6 (Sirt6) in microglia and the deacetylation of H3 lysine9 (H3K9ac) by Sirt6, thereby reducing the expression of H3K9ac. This process can reduce the degree of neuroinflammation in diabetic mice, reduce iron accumulation caused by pro-inflammatory reaction, and further inhibit ferroptosis (Wang Y. et al., 2021).

#### 5.3.4 Protein Glycosylation

The role of glycosylation modification of proteins in ferroptosis may not be emphatically discussed. During cell transformation, the main types of protein glycosylation changes are O-glycan (GalNAc-Ser/Thr) and N-glycan (Rodrigues et al., 2018). The down regulation of GALNT14, which is up-regulated in ovarian cancer, inhibits the activity of mTOR pathway through O-glycosylation of EGFR, thereby inhibiting ferroptosis in ovarian cancer cells (Lee et al., 2020). Lysosome-associated membrane protein 2 (LAMP2) is a highly glycosylated lysosome membrane protein. E participating in the autophagy process of cells, LAMP2 can also affect the process of ferroptosis. New researches have uncovered that the lack of LAMP2 is in connected with ROS. LAMP2—KD (LAMP knock-down) reduces the concentration of cytosolic cysteine, while cysteine starvation can lead to decreased antioxidant capacity and mitochondrial lipid peroxidation. Supplementation of cysteine can restore GSH and prevent cell death caused by LAMP knockdown (Lee et al., 2020).

## 6 Conclusion

The principal axis of ferroptosis mechanism is as the sequence, iron accumulation, ROS accumulation and then lipid peroxidation. It involves the metabolic process of various substances and the participation of different organelles. The general pathway to inhibit ferroptosis is how to cut the spindle before the toxicity of cells cannot be reversed, or to reverse the harm caused as much as possible by supplementing raw materials or reducing components. In different diseases, the treatment methods targeting ferroptosis can also be different. The up-regulation of ferroptosis in tumors will help reduce cancer cells (Chen X. et al., 2021), and for diseases such as ischemia reperfusion, ferroptosis needs to be avoided as much as possible (Lillo-Moya et al., 2021). Among the three main metabolic pathways, the accumulation of iron in the central ganglia and the overloaded lipid peroxides promote ferroptosis (directing oxidation), while amino acids (represented by cysteine) play a role in reduction and remediation. Among the metabolic of different substances, how to influence the synergy and antagonism forces of ferroptosis above in order to achieve the desired results of treatment, will be an interesting topic.

Ferroptosis is a programmed cell death process involving cell lipid metabolism, ROS production and intracellular LIP changes. With the passive participation of organelles, these messes finally guide to the cell membrane rupture. At present, we have got somewhere in the molecular occurrence and regulation mechanism of ferroptosis. However, it remains other questions to be figured out. What role does mitochondrial metabolic process play in ferroptosis-related signaling pathways, and whether the related pathways in mitochondria can be used as targets of future drug researches and developments. Whether balance of the lipid metabolism can be maintained by reducing the occurrence of endoplasmic reticulum stress, and the results can further adjust the endoplasmic reticulum-mediated intercellular communication process. in addition, as to the study of organelles, how to design molecular probes in order to further study the changes of Golgi apparatus in the process of ferroptosis, and how to understand the role of lysosomes in autophagy and ferroptosis-whether they affect each other or are independent of each other. These questions still need further research to answer.

Overall, the regulatory network of ferroptosis can be roughly divided into two interrelated directions-non-epigenetic methods, dependant on metabolism and regulation of various factors, and the other is the epigenetic methods. Epigenetics involves a wide range and details, for example, its modification can involve all aspects of translation-The structural adjustment of the chromatin as a whole, the micro-regulation of DNA and RNA, and the influence of all aspects of post-translational modification downstream of the central law. Meanwhile, the mechanisms and methods of modification are not monotonous-different perssads can participate in the modification, and non-coding RNA can regulate each process under different conditions. The links between different steps make it a huge network. At the same level, different ways of post-translational modification are related and influenced mutually. Distinct epigenetic factors also maintain at different levels within the same pathway, such as transcription, translation and modification (Dai et al., 2020). These phenomena can describe how epigenetic regulation creates a complex and extensive delicate balance. And whether there are other regulatory mechanisms in this course, and how the relationships is like between epigenetic and non-epigenetic patterns, are also the research about the epigenetic regulations of ferroptosis that needs to be explored further in the future.

In addition, at the level of post-translational modification, its modifying targets are often divided into histone and non-histone (Yang et al., 2021), and the former has received more attention than the latter due to its closer relationship with genetic materials. It can be predicted that how epigenetic regulators affect non-histone proteins is a complex area of exploration. Similarly, the glycosylation modification is rarely mentioned. It is also a future direction that whether there are any other pathways to guide ferroptosis and whether they have the same status as important modification methods such as ubiquitination.

Finally, the regulatory factor network of ferroptosis, the unknown downstream targets and other details are not clear, but it will undoubtedly provide us with more new treatment directions a new perspective different from the previous cell death network and more new treatment directions, which will motivate new research, and the new technology of curing ferroptosis-related diseases.
